# Analysis of data from two influenza surveillance hospitals in Zhejiang province, China, for the period 2018–2022

**DOI:** 10.1371/journal.pone.0299488

**Published:** 2024-02-28

**Authors:** Yuda Wang, Yan Liu, Guangtao Liu, Xiuxiu Sun, Zizhe Zhang, Jianyong Shen

**Affiliations:** Huzhou Center for Disease Control and Prevention, Huzhou, Zhejiang, China; Haute Autorite de sante, FRANCE

## Abstract

**Purpose:**

To assess the epidemiology of seasonal influenza in Huzhou City, Zhejiang Province, China, during 2018–2022 and provide insights for influenza prevention.

**Methods:**

Following the National Influenza Surveillance Program, we conducted pathogen surveillance by randomly sampling throat swabs from cases with influenza-like illness (ILI) at two sentinel hospitals.

**Results:**

From 2018 to 2022, a total of 3,813,471 cases were treated at two hospitals in Huzhou, China. Among them, there were 112,385 cases of Influenza-Like Illness (ILI), accounting for 2.95% of the total number of cases. A total of 11,686 ILI throat swab samples were tested for influenza viruses, with 1,602 cases testing positive for influenza virus nucleic acid, resulting in a positivity rate of 13.71%. Among the positive strains, there were 677 strains of A(H3N2) virus, 301 strains of A(H1N1) virus, 570 strains of B/Victoria virus, and 54 strains of B/Yamagata virus. The ILI percentage (ILI%) and influenza nucleic acid positivity rate showed winter-spring peaks in the years 2018, 2019, 2021, and 2022, with the peaks concentrated in January and February. Additionally, a small peak was observed in August 2022 during the summer season. No peak was observed during the winter-spring season of 2020. The highest proportion of ILI cases was observed in children aged 0–4 years, followed by school-age children aged 5–14 years. There was a positive correlation between ILI% and influenza virus nucleic acid positivity rate (r = 0.60, p < 0.05).

**Conclusions:**

The influenza outbreak in Huzhou from 2020 to 2022 was to some extent influenced by the COVID-19 pandemic and public health measures. After the conclusion of the COVID-19 pandemic, the influenza outbreak in Huzhou may become more severe. Therefore, it is crucial to promptly assess the influenza outbreak trends based on the ILI% and the positivity rate of influenza virus nucleic acid tests.

## Introduction

Influenza, commonly known as the flu, is one of the most contagious and widely spread respiratory diseases in the world. It is highly prone to causing epidemics and outbreaks and is the first infectious disease to be globally monitored by the World Health Organization (WHO) [1–3]. Globally, approximately 1 billion people are affected by the flu each year, with 290,000 to 650,000 individuals dying from flu-related illnesses. In China, over 88,000 people succumb to the flu annually [4,5], making it a significant public health burden in the country. The influenza virus belongs to the Orthomyxoviridae family and is a single-stranded, negative-sense, segmented RNA virus. The main subtypes of the influenza virus include Type A with H1N1 (A/H1N1) and H3N2 (A/H3N2) subtypes, as well as Type B with Victoria lineage (BV) and Yamagata lineage (BY) influenza viruses [6–8]. Influenza surveillance plays a vital role in early detection of flu outbreaks. China established its National Influenza Surveillance Center in 1957 and has since developed a comprehensive monitoring network [9,10].

The general population is universally susceptible to the influenza virus, primarily due to the virus’s tendency for antigenic drift. Antigenic drift involves genetic mutations in the influenza virus, making it challenging for the human body to mount effective immune responses [11]. Because of this readily occurring genetic variability, interrupting the transmission of the influenza virus becomes exceedingly difficult [12]. The prevalence of influenza also cannot be precisely predicted, adding complexity to determining when large-scale outbreaks might occur. Consequently, influenza poses a significant threat to human health. Given the widespread susceptibility of the population, influenza spreads rapidly in society, posing a substantial challenge to public health. Despite concerted efforts by scientists and healthcare professionals through influenza vaccines and preventive measures, controlling influenza remains challenging due to the high variability of the influenza virus [13–15]. The impact of influenza is not limited to the direct incidence of diseases and deaths but extends to its effects on the functioning of society and the economy. Influenza outbreaks can result in a significant number of work absences and strain on healthcare resources, placing considerable pressure on various industries and communities [16,17].

This study analyzes the data collected by the influenza surveillance system in Huzhou, Zhejiang Province, China, from 2018 to 2022, with the aim of understanding the characteristics of influenza prevalence over the five-year period and providing reference data for the formulation of influenza prevention and control strategies.

## Materials and methods

### Influenza-like illness (ILI) surveillance

The data used in this study were obtained from the surveillance of Influenza-Like Illness (ILI) cases conducted by the Huzhou City National Influenza Surveillance Network from 2018 to 2022, as well as data from pathogen surveillance. The Huzhou City National Influenza Surveillance Network comprises two influenza surveillance sentinel hospitals and one influenza network laboratory. These two sentinel hospitals are the Huzhou City First People’s Hospital, located in Wuxing District, and the Huzhou City Nanxun District People’s Hospital, situated in Nanxun District. The network laboratory is operated by the Huzhou City Center for Disease Control and Prevention.The definition of outpatient and emergency department cases is as follows: it includes all patients seen in the internal medicine outpatient, internal medicine emergency, fever outpatient, and/or pediatric internal medicine outpatient and emergency departments of the hospital. ILI is defined as cases with a body temperature of ≥38°C accompanied by either cough or sore throat. The total number of ILI and outpatient and emergency department cases is consistent in each monitoring department. Throat swabs from ILI cases occurring within 3 days of symptom onset were collected for influenza virus testing, and all patients did not use antiviral medications. The hospital ILI percentage (ILI%) represents the proportion of ILI cases among the total outpatient and emergency department cases in the hospital. Data entry for ILI% is carried out by staff at the sentinel hospitals and specimen information for the cases is entered into the influenza monitoring system by the Huzhou City Center for Disease Control and Prevention. Raw data are shown in S1 and S2 Tables.

### Influenza pathogen surveillance

The virus RNA was extracted using the fully automatic nucleic acid extractor from Xi’an Tianlong Technology Co., Ltd. (Model: NP968) along with its matching extraction reagents. The influenza virus nucleic acid detection was carried out using the quadruple real-time fluorescent PCR detection kit for influenza A virus/influenza B virus/adenovirus/respiratory syncytial virus (Zhuochenghuisheng, China, A7034YH-50T) and the double real-time fluorescent PCR detection kit for the nucleic acid of influenza A H1N1 subtype/seasonal H3 subtype of human influenza virus (Zhuochenghuisheng, China, A3702YH-50T). All procedures were strictly conducted according to the instrument operating procedures and reagent kit instructions.

### Quality control

Personnel at the sentinel hospitals have received specialized training and strictly adhere to the requirements of the national influenza monitoring protocol. Designated individuals are responsible for case information collection and specimen collection. The Huzhou City Center for Disease Control and Prevention regularly conducts supervisory inspections of the influenza monitoring processes at sentinel hospitals and ensures that any identified issues are promptly rectified. The influenza network laboratory is a nationally accredited laboratory, and influenza virus nucleic acid positive samples are sent to the Zhejiang Provincial Center for Disease Control and Prevention for confirmation.

### Statistical analysis

A database is established using Excel 2010, and statistical analysis is conducted using SPSS 25.0 software. The significance level is set at a two-tailed alpha (α) of 0.05 for trend analysis.

## Results

### General characteristics of ILI cases

From 2018 to 2022, the Huzhou City First People’s Hospital had a total of 2,561,620 outpatient cases, with 89,421 of them being ILI cases, representing an ILI% of 3.49%. The Nanxun District People’s Hospital had a total of 1,251,851 outpatient cases, with 22,964 of them being ILI cases, resulting in an ILI% of 1.83%. Over the same period, the total outpatient cases at the influenza monitoring points in Huzhou City amounted to 3,813,471, with 112,385 of them being ILI cases and an ILI% of 2.95%. The ILI percentages for the years 2018 to 2022 were 3.32%, 3.85%, 2.76%, 1.95%, and 2.71% respectively. The highest ILI% was in 2019, and the lowest was in 2021. Overall, there is a fluctuating downward trend. (*χ*^2^ = 2724.21, P<0.001). Refer to Table 1.

**Table 1:** ILI cases and ILI% at monitoring points in Huzhou City, Zhejiang Province, 2018–2022.

Year	Huzhou City First People’s Hospital			Huzhou City Nanxun District People’s Hospital			Total		
	Number of ILI cases	Total number of outpatient cases	ILI%	Number of ILI cases	Total number of outpatient cases	ILI%	Number of ILI cases	Total number of outpatient cases	ILI%
2018	24525	541705	4.53	2330	266671	0.87	26855	808376	3.32
2019	29163	576456	5.06	3013	258333	1.17	32176	834789	3.85
2020	13744	405425	3.39	3611	223560	1.62	17355	628985	2.76
2021	9266	509028	1.82	5596	253558	2.21	14862	762586	1.95
2022	12723	529006	2.41	8414	249729	3.37	21137	778735	2.71
Total	89421	2561620	3.49	22964	1251851	1.83	112385	3813471	2.95

### Age distribution characteristics of ILI cases

From 2018 to 2022, ILI cases occurred in all age groups, and the composition of cases in different age groups was similar in each epidemic year. Cases were primarily concentrated in those aged under 5, accounting for 53.36% of the total cases. This proportion showed a decreasing trend (*χ*^2^ = 5309.47, P<0.001). The next highest group of cases was in the 5–14 age group, representing 29.61% of the total cases, with an increasing trend in this proportion (*χ*^2^ = 853.19, P<0.001). The combined proportion of cases aged 14 and below was 82.96%. The age groups 15–24, 25–59, and 60 and above accounted for 4.46%, 10.43%, and 2.14%, respectively. Refer to Table 2.

**Table 2:** Number of ILI cases and distribution by age groups in Huzhou City, Zhejiang Province, 2018–2022.

Year	ILI case counts by age group (as a percentage of total ILI cases, %)				
	0~	5~	15~	25~	60~
2018	18317(68.21)	6609(24.61)	494(1.84)	1099 (4.09)	336 (1.25)
2019	19715 (61.27)	9115 (28.33)	735 (2.28)	2155 (6.70)	456(1.42)
2020	6461 (37.23)	5072(29.23)	1933(11.14)	3220 (18.55)	669(3.85)
2021	7413 (49.88)	4728(31.81)	618 (4.16)	1753(11.80)	350(2.35)
2022	8051(38.09)	7755(36.39)	1234 (5.84)	3499(16.55)	598(2.83)
Total	5995 (53.36)	33279 (29.61)	5014 (4.46)	11726 (10.43)	2409 (2.14)
*χ*^*2*^ value	5309.47	853.19	708.89	2527.96	220.143
*P* value	0.000	0.000	0.000	0.000	0.000

### Seasonal distribution characteristics of ILI

**Fig 1:**
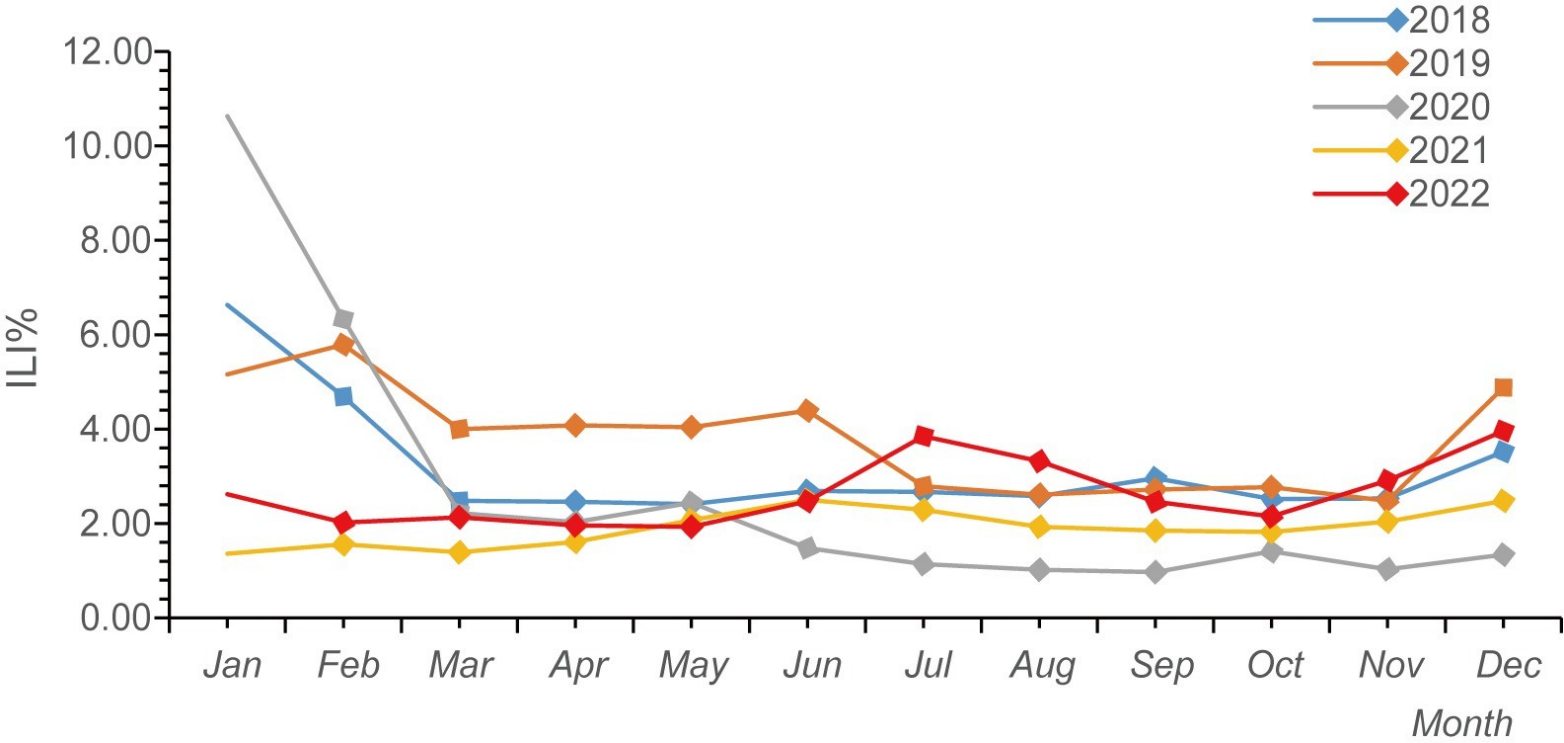
Distribution of ILI% in different months in Huzhou City, Zhejiang Province, from 2018 to 2022.

There were winter-spring peaks in 2018, 2019, 2021, and 2022, concentrated in January and February. In this study, "winter-spring season" refers to November of the current year through March of the following year. Additionally, a small summer peak occurred in August 2022. No winter-spring peak was observed in 2020. The ILI% fluctuated in the range of 0.97% to 10.63% each month. The average ILI% for January across all years was the highest at 5.28%, while October had the lowest at 2.13%. Specifically, in January 2020, the ILI% was the highest among all months over the five-year period, reaching 10.63% (Fig 1, S3 Table).

### General characteristics of influenza epidemics

From 2018 to 2022, a total of 11,686 throat swab specimens from ILI cases were collected, and 1,602 influenza virus positives were detected, with a positivity rate of 13.71%. The positivity rates for the respective years from 2018 to 2022 were 11.07%, 14.38%, 5.66%, 7.70%, and 28.62%. Among all positive strains, there were 677 strains of A(H3N2) virus, accounting for 42.26%, 301 strains of A(H1N1) virus, accounting for 18.79%, 570 strains of B/Victoria lineage, accounting for 35.58%, and 54 strains of B/Yamagata lineage, accounting for 3.37%.

**Fig 2:**
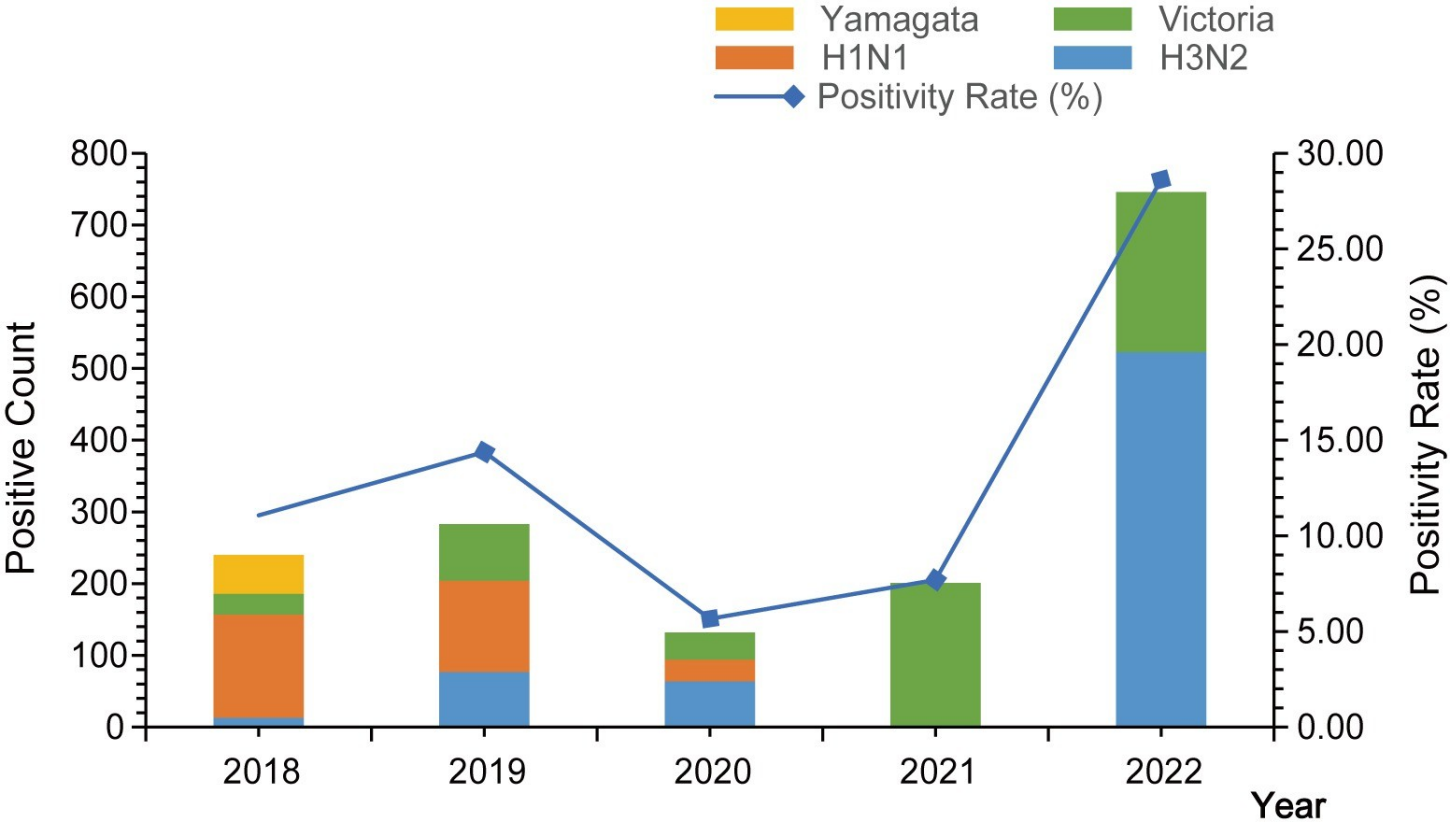
Distribution of different types of influenza viruses in Huzhou City, Zhejiang Province, from 2018 to 2022.

From 2018 to 2020, A(H1N1), A(H3N2), and B/Victoria lineage viruses co-circulated, with A(H1N1) virus being the dominant strain. Only 54 cases of B/Yamagata lineage virus were detected in 2018, and no cases were detected in other years. In 2021, B/Victoria lineage was the dominant circulating strain, while in 2022, A(H3N2) and B/Victoria lineage viruses co-circulated, with A(H3N2) being the dominant strain. A(H1N1) virus was not detected for two consecutive years in 2021 and 2022 (Fig 2, S4 Table).

### Seasonal distribution characteristics of influenza positive strains

**Fig 3:**
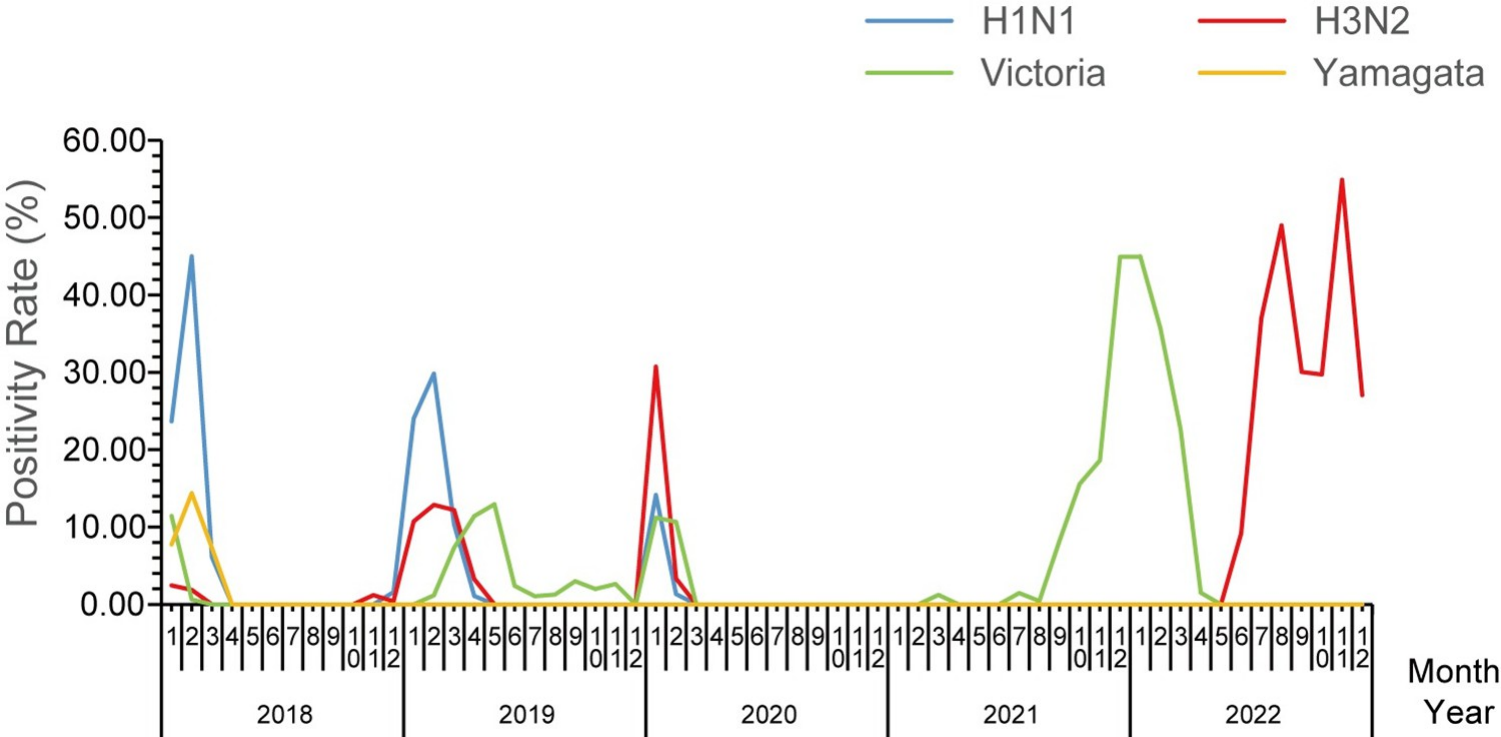
Monthly distribution of influenza positivity rates for different types in Huzhou City, Zhejiang Province, from 2018 to 2022.

In the years 2018, 2019, and 2021, the influenza epidemic was mainly concentrated from December to February of the following year, with peaks occurring each year in January and February. In the winter-spring season of 2020, there was no influenza epidemic. In 2022, the influenza epidemic occurred earlier, starting in July, with two peak seasons in both summer and winter-spring, reaching peaks in August and November, respectively. In February 2018, the highest positivity rate was 62.47%; in February 2019, it was 43.94%; in January 2020, it was 58.25%. The positivity rates for August and November 2022 were 48.40% and 55.71%, respectively (Fig 3, S5 Table).

### Influenza positive strain distribution characteristics across different age groups

From 2018 to 2022, influenza positive strains occurred in various age groups, with the proportions of positive strains in different age groups being 14.29%, 40.20%, 11.05%, 30.21%, and 4.25%, respectively. The age group 5–14 had the highest proportion, while the age group 60 and above had the lowest. Among them, A(H3N2) and B(Victoria) strains had the highest proportion in the 5–14 age group, while A(H1N1) and B(Yamagata) had the highest proportion in the 25–59 age group. There were differences in the proportions of positive strains across different age groups (χ^2^ = 105.32, p < 0.001). Refer to Table 3.

**Table 3:** Distribution of influenza positive strains across different age groups in Huzhou City, Zhejiang Province.

Virus Typing	Number of Positives in Each Age Group (Positivity Composition Ratio)				
	0~	5~	15~	25~	60~
A (H3N2)	89(13.15)	315(46.53)	91(13.44)	145(21.42)	37(5.46)
A (H1N1)	53(17.61)	71(23.59)	25(8.31)	133(44.19)	19(6.30)
B (Victoria)	77(13.51)	247(43.33)	53(9.30)	186(32.63)	7(1.23)
B (Yamagata)	10(18.52)	11(20.37)	8(14.81)	20(37.04)	5(9.26)
Total	229(14.29)	644(40.20)	177(11.05)	484(30.21)	68(4.25)
χ2 value	105.32				
P value	<0.001				

### Relationship between ILI and influenza virus nucleic acid positivity

**Fig 4:**
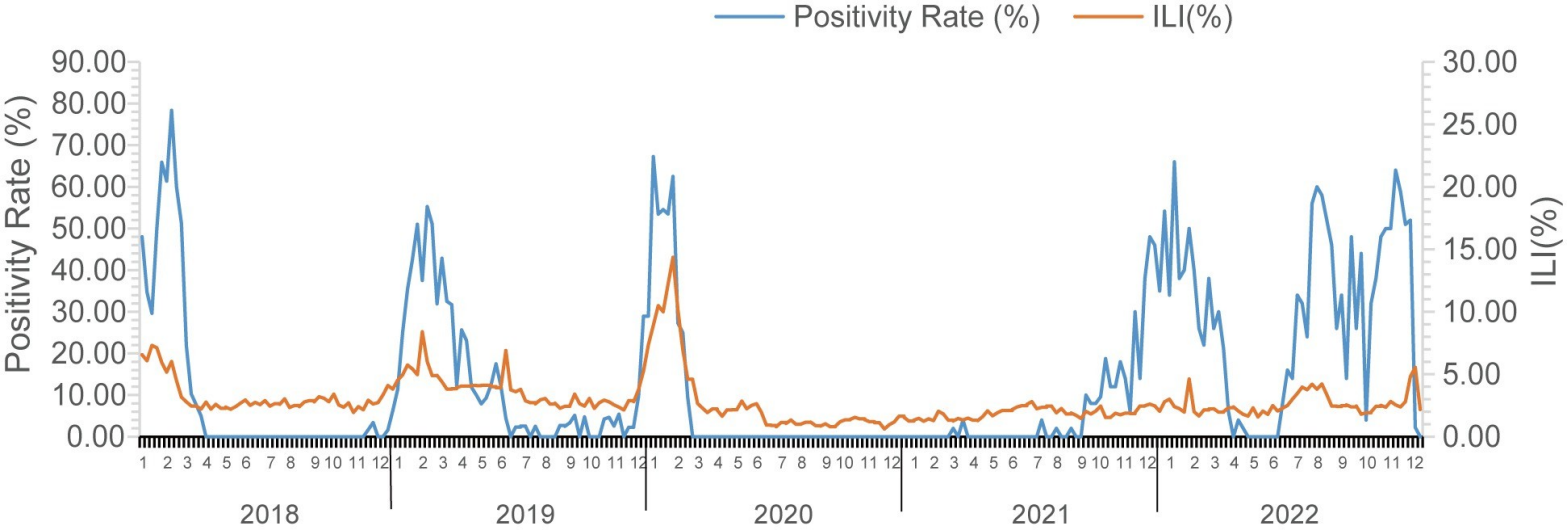
The relationship between influenza ILI% (Influenza-Like Illness percentage) and influenza Positivity Rate (%) from 2018 to 2022.

Correlation analysis was conducted between the weekly ILI% and sample influenza virus nucleic acid positivity rates from 2018 to 2022. There was a correlation between ILI% and the sample influenza virus nucleic acid positivity rate, with a correlation coefficient (r) of 0.60, and the difference was statistically significant (P<0.001). Refer to Fig 4 and S6 Table.

## Discussion

From 2018 to 2022, Huzhou City’s ILI% showed a fluctuating downward trend, and the positivity rate for influenza in 2020–2021 also significantly decreased compared to previous years. This aligns with the substantial reduction in global influenza activity after 2020, following the outbreak of the COVID-19 pandemic, where public health measures and travel restrictions severely suppressed global influenza transmission [18,19]. During this period, Huzhou City also implemented strict epidemic screening and control measures. On one hand, it standardized fever outpatient and pre-check triage, focusing on testing for COVID-19 nucleic acid, which may have led to a decrease in the positivity rate for influenza virus. On the other hand, measures such as widespread mask-wearing, maintaining effective social distancing, and reducing gatherings also contributed to a reduction in the transmission of respiratory infectious diseases, including influenza.

The exceptionally high ILI% in Huzhou in January 2020 was attributed to a decreased willingness of the public to seek medical attention after the implementation of control measures. This resulted in a significant reduction in the total number of outpatient and emergency department visits, the denominator for ILI%, rather than an increase in the number of influenza-like cases. Studies indicate that the typical flu season in the Northern Hemisphere starts in October, with peak activity occurring in January or February [20]. China experiences seasonal peaks in winter and spring, with intermittent peaks in the summer [21]. In this study, Huzhou City exhibited winter-spring peaks in 2018, 2019, and 2021. Notably, there was no winter-spring peak from late 2020 to early 2021. This was the first time in over a decade, as reported by LIU’s study on influenza monitoring in Huzhou City from 2011 to 2017 [22], that a winter-spring flu season did not occur. This further demonstrates the effectiveness of Huzhou’s COVID-19 control measures. The occurrence of both summer and winter-spring peaks in Huzhou in 2022 suggests that in the coming years, the city needs to be prepared for influenza prevention and control during both seasons. This could be attributed to the gradual relaxation of public health measures in 2022, coupled with an increased willingness of people to seek medical attention. As a result, the positivity rate of influenza virus nucleic acid tests has significantly risen, indicating the need for ongoing vigilance beyond the COVID-19 pandemic [23,24].

Influenza viruses exhibit high variability, leading to changes in dominant strains from year to year. In Huzhou City from 2018 to 2022, A(H3N2), A(H1N1), and B/Victoria strains alternated as dominant strains, consistent with the monitoring of prevalent strains by the Chinese National Influenza Center [25,26]. B/Yamagata strain was only detected in 2018, and for the subsequent four years, it was not found. This aligns with WHO reports indicating a global absence of B/Yamagata lineage influenza viruses for three years since March 2020. Studies suggest weakened antigenic selection for B/Yamagata strains before the COVID-19 pandemic, leading to a further decline in prevalence over time, possibly resulting in the disappearance of B/Yamagata strains globally [19,27]. However, treating B/Yamagata as a high-consequence pathogen is necessary to prevent its resurgence.

Children and adolescents are particularly vulnerable to influenza due to their immature immune systems and weaker resistance [2]. In this study, the proportion of ILI cases was highest in children aged 0–4 years, followed by school-age children aged 5–14 years, with an increasing trend in the latter age group. The positivity rate for influenza was mainly observed in school-age children aged 5–14 years. Similar findings were reported in studies from Ningbo City, Guizhou Province, and others [28–32]. The extended gathering time of school-age children in schools makes them more prone to influenza transmission. Once symptoms appear, parents tend to seek medical attention promptly, leading to a higher ILI proportion [33]. Notably, the study suggests that influenza vaccination rates are rising among individuals aged 60 and above in Huzhou due to the implementation of influenza vaccination programs. However, the influenza vaccination rate among children remains very low. This highlights the need for schools to actively organize influenza vaccination for students and promptly handle and report clustered vaccine-related incidents. Students should also follow preventive measures such as wearing masks, frequent handwashing, and ensuring good ventilation.

The study demonstrates a correlation between nucleic acid test results and ILI monitoring, indicating that ILI% and influenza positivity rates are positively correlated. As ILI% provides a quicker and more comprehensive snapshot compared to influenza virus nucleic acid testing, changes in ILI% can serve as an early indicator of influenza trends, offering significant value in influenza surveillance and forecasting.

In conclusion, the current patterns of influenza transmission have been influenced to some extent by the COVID-19 pandemic and its control measures. With the implementation of COVID-19 prevention and control measures, subsequent trends in the prevalence of COVID-19, influenza, and other respiratory infectious diseases may also change. It is crucial to strengthen ongoing monitoring and analysis. Due to limitations in facilities at two hospitals and the Huzhou City Center for Disease Control and Prevention, this study did not conduct subsequent virus identification and gene sequencing and resistance analysis. Future research will delve into these aspects for a more in-depth understanding of influenza viruses.

## Supporting information

S1 Table Reporting data from sentinel hospital networks for Week 1 of 2018 to Week 52 of 2022. (XLSX)

S2 Table Pathological analysis data of influenza-like cases in Huzhou City, Zhejiang Province, China, from 2018 to 2022. (XLSX)

S3 Table Distribution data of ILI% in Huzhou City, Zhejiang Province, China, for different months from 2018 to 2022. (XLSX)

S4 Table Distribution data of different types of influenza viruses in Huzhou City, Zhejiang Province, China, from 2018 to 2022. (XLSX)

S5 Table Monthly distribution data of influenza positivity rates for different types in Huzhou City, Zhejiang Province, China, from 2018 to 2022. (XLSX)

S6 Table Data on Influenza-like Illness Percentage (ILI%) and Influenza Positivity Rate (%) from 2018 to 2022. (XLSX)

## References

[1] Brody H. Influenza. Nature. 2019;573(7774):S49. doi: 10.1038/d41586-019-02750-x 31534258

[2] Gaitonde DY, Moore FC, Morgan MK. Influenza: Diagnosis and Treatment. Am Fam Physician. 2019;100(12):751–758. 31845781

[3] Salvador García C, Gimeno Cardona C. Influenza: A preventable infection in different populations. Enferm Infecc Microbiol Clin (Engl Ed). 2023;41(7):387–390. doi: 10.1016/j.eimce.2023.05.006 37394402

[4] Dai P, Wang Q, Jia M, Leng Z, Xie S, Feng L, et al. Driving more WHO-recommended vaccines in the National Immunization Program: Issues and challenges in China. Hum Vaccin Immunother. 2023;19(1):2194190. doi: 10.1080/21645515.2023.2194190 37099400 PMC10158540

[5] Wang X, Fan Y, Wang W. Investigation of non-National Immunization Program vaccination intentions in rural areas of China. BMC Public Health. 2023;23(1):1485. doi: 10.1186/s12889-023-16390-4 37542236 PMC10401748

[6] Park JE, Ryu Y. Transmissibility and severity of influenza virus by subtype. Infect Genet Evol. 2018;65:288–292. doi: 10.1016/j.meegid.2018.08.007 30103034

[7] Te Velthuis AJ, Fodor E. Influenza virus RNA polymerase: insights into the mechanisms of viral RNA synthesis. Nat Rev Microbiol. 2016;14(8):479–93. doi: 10.1038/nrmicro.2016.87 27396566 PMC4966622

[8] Skelton RM, Huber VC. >Comparing Influenza Virus Biology for Understanding Influenza D Virus. Viruses. 2022;14(5):1036. doi: 10.3390/v14051036 35632777 PMC9147167

[9] Wang DY. Development and prospect of Influenza Surveillance Network in China. Zhonghua Liu Xing Bing Xue Za Zhi. 2018;39(8):1036–1040. doi: 10.3760/cma.j.issn.0254-6450.2018.08.005 30180424

[10] Huang WJ, Cheng YH, Tan MJ, Liu J, Li XY, Zeng XX, et al. Epidemiological and virological surveillance of influenza viruses in China during 2020–2021. Infect Dis Poverty. 2022;11(1):74. doi: 10.1186/s40249-022-01002-x 35768826 PMC9244124

[11] Yewdell JW. Antigenic drift: Understanding COVID-19. Immunity. 2021;54(12):2681–2687. doi: 10.1016/j.immuni.2021.11.016 34910934 PMC8669911

[12] Peteranderl C, Herold S, Schmoldt C. Human Influenza Virus Infections. Semin Respir Crit Care Med. 2016;37(4):487–500. doi: 10.1055/s-0036-1584801 27486731 PMC7174870

[13] El Ramahi R, Freifeld A. Epidemiology, Diagnosis, Treatment, and Prevention of Influenza Infection in Oncology Patients. J Oncol Pract. 2019;15(4):177–184. doi: 10.1200/JOP.18.00567 30970229

[14] Sullivan SJ, Jacobson RM, Dowdle WR, Poland GA. 2009 H1N1 influenza. Mayo Clin Proc. 2010;85(1):64–76. doi: 10.4065/mcp.2009.0588 20007905 PMC2800287

[15] O’Driscoll LS, Martin-Loeches I. Management of Severe Influenza. Semin Respir Crit Care Med. 2021;42(6):771–787. doi: 10.1055/s-0041-1735491 34918320

[16] Tang RB, Chen HL. An overview of the recent outbreaks of the avian-origin influenza A (H7N9) virus in the human. J Chin Med Assoc. 2013;76(5):245–8. doi: 10.1016/j.jcma.2013.04.003 23651506

[17] Webster RG, Govorkova EA. Continuing challenges in influenza. Ann N Y Acad Sci. 2014;1323(1):115–39. doi: 10.1111/nyas.12462 24891213 PMC4159436

[18] Javanian M, Barary M, Ghebrehewet S, Koppolu V, Vasigala V, Ebrahimpour S. A brief review of influenza virus infection. J Med Virol. 2021;93(8):4638–4646. doi: 10.1002/jmv.26990 33792930

[19] Dhanasekaran V, Sullivan S, Edwards KM, Xie R, Khvorov A, Valkenburg SA, et al. Human seasonal influenza under COVID-19 and the potential consequences of influenza lineage elimination. Nat Commun. 2022;13(1):1721. doi: 10.1038/s41467-022-29402-5 35361789 PMC8971476

[20] Umuhoza T, Bulimo WD, Oyugi J, Schnabel D, Mancuso JD. Prevalence and factors influencing the distribution of influenza viruses in Kenya: Seven-year hospital-based surveillance of influenza-like illness (2007–2013). PLoS One. 2020;15(8):e0237857. doi: 10.1371/journal.pone.0237857 32822390 PMC7446924

[21] Chen C, Liu X, Yan D, Zhou Y, Ding C, Chen L, et al. Global influenza vaccination rates and factors associated with influenza vaccination. Int J Infect Dis. 2022;125:153–163. doi: 10.1016/j.ijid.2022.10.038 36328290

[22] Yan Liu, Jianyong Shen, Dong Wen, Guangtao Liu. Influenza surveillance in Huzhou during 2011–2017. Shanghai Journal of Preventive Medicine. 2021; 33(2):115–119. doi: 10.19428/j.cnki.sjpm.2021.19290

[23] Chotpitayasunondh T, Fischer TK, Heraud JM, Hurt AC, Monto AS, Osterhaus A, et al. Influenza and COVID-19: What does co-existence mean? Influenza Other Respir Viruses. 2021;15(3):407–412. doi: 10.1111/irv.12824 33128444 PMC8051702

[24] Czubak J, Stolarczyk K, Orzeł A, Frączek M, Zatoński T. Comparison of the clinical differences between COVID-19, SARS, influenza, and the common cold: A systematic literature review. Adv Clin Exp Med. 2021;30(1):109–114. doi: 10.17219/acem/129573 33529514

[25] Yang R, Sun H, Gao F, Luo K, Huang Z, Tong Q, et al. Human infection of avian influenza A H3N8 virus and the viral origins: a descriptive study. Lancet Microbe. 2022;3(11):e824–e834. doi: 10.1016/S2666-5247(22)00192-6 36115379

[26] Wu M, Su R, Gu Y, Yu Y, Li S, Sun H, et al. Molecular Characteristics, Antigenicity, Pathogenicity, and Zoonotic Potential of a H3N2 Canine Influenza Virus Currently Circulating in South China. Front Microbiol. 2021;12:628979. doi: 10.3389/fmicb.2021.628979 33767679 PMC7985081

[27] Park JE, Ryu Y. Transmissibility and severity of influenza virus by subtype. Infect Genet Evol. 2018;65:288–292. doi: 10.1016/j.meegid.2018.08.007 30103034

[28] Wan YH, Zhuang L, Zheng QN, Ren LJ, Fu L, Jiang WJ, et al. Genetic characteristics of hemagglutinin and neuraminidase of avian influenza A (H7N9) virus in Guizhou province, 2014–2017. Zhonghua Liu Xing Bing Xue Za Zhi. 2018;39(11):1465–1471. doi: 10.3760/cma.j.issn.0254-6450.2018.11.009 30462955

[29] Zhou Y, Wang Y, Cheng J, Zhao X, Liang Y, Wu J. Molecular epidemiology and antimicrobial resistance of *Haemophilus influenzae* in Guiyang, Guizhou, China. Front Public Health. 2022;10:947051. doi: 10.3389/fpubh.2022.947051 36530676 PMC9751421

[30] Hu FJ, Li YD, Jiao SL, Zhang S. Genetic variation of the hemagglutinin and neuraminidase of influenza B viruses isolated in Ningbo during 2010–2012. Zhonghua Yu Fang Yi Xue Za Zhi. 2013;47(12):1100–4. 24529267

[31] Zheng S, Zou Q, Wang X, Bao J, Yu F, Guo F, et al. Factors Associated With Fatality Due to Avian Influenza A(H7N9) Infection in China. Clin Infect Dis. 2020;71(1):128–132. doi: 10.1093/cid/ciz779 31418813 PMC8127055

[32] Wang XX, Cheng W, Yu Z, Liu SL, Mao HY, Chen EF. Risk factors for avian influenza virus in backyard poultry flocks and environments in Zhejiang Province, China: a cross-sectional study. Infect Dis Poverty. 2018;7(1):65. doi: 10.1186/s40249-018-0445-0 29914558 PMC6006748

[33] Grohskopf LA, Blanton LH, Ferdinands JM, Chung JR, Broder KR, Talbot HK, et al. Prevention and Control of Seasonal Influenza with Vaccines: Recommendations of the Advisory Committee on Immunization Practices—United States, 2022–23 Influenza Season. MMWR Recomm Rep. 2022;71(1):1–28. doi: 10.15585/mmwr.rr7101a1 36006864 PMC9429824

